# Retraction Note: Early diagnosis of oral cancer using a hybrid arrangement of deep belief networkand combined group teaching algorithm

**DOI:** 10.1038/s41598-025-22725-5

**Published:** 2025-10-07

**Authors:** Wenjing Wang, Yi Liu, Jianan Wu

**Affiliations:** 1 Department of Stomatology, The First Affiliated Hospital of Yangtze University, Jingzhou, 434000 Hubei China; 2 Experimental and Practical Teaching Center, Hubei College of Chinese Medicine, Jingzhou, 434000 Hubei China

Retraction of: *Scientific Reports* 10.1038/s41598-023-49438-x, published online 12 December 2023

The Editors have retracted this Article.

After publication of this paper, concerns were raised about the relevance of certain references to this study. The Authors did not respond to these concerns, nor to a request to send the underlying code and data. The Editors therefore lost confidence in the findings reported in this Article.

All Authors agree with the retraction and its wording.

